# Supplementary material for: Plasmonic computing of spatial differentiation
Source: Nat Commun. 2017 May 19;8:15391. doi: 10.1038/ncomms15391 (PMC5454537; doi:10.1038/ncomms15391)
Supplement: Supplementary Information — Supplementary Notes, Supplementary Figures and Supplementary References [file ncomms15391-s1.pdf]

Supplementary Note 1: Spatial transfer function for surface plasmon polariton (SPP) excitations at a metallic surface

To analyze the the spatial transfer function, we developed the spatial coupled-mode theory to describe the SPP excitation at a metallic surface. As Supplementary Fig. 1 shows, in the Kretschmann configuration, a p-polarized incident beam illuminates the metal layer coating on a glass prism. When the parallel component of the incident wavevector, $\tilde{k}_z$, is close to the SPP wavevector β_{spp}, the incident light excites an SPP through an evanescent wave. Meanwhile the excited SPP leaks out and generates the radiation wave in the glass as it propagates along the $\tilde{z}$ direction. Therefore, the reflection process consists of two pathways: the direct reflectance of the incident wave at the glass-metal interface, and the outgoing radiation from the leakage of the excited SPP at the metal-air interface. Based on the spatial coupled-mode theory [1, 2], the spatial mode coupling and the interference process is described by the following equations:

$$\frac{da}{d\tilde{z}} = (i\beta_{\mathrm{spp}} - \alpha_{\mathrm{l}} - \alpha_{\mathrm{spp}})a + ie^{i\phi/2}\sqrt{2\alpha_{\mathrm{l}}}\tilde{S}_{\mathrm{in}}(\tilde{z}) \tag{1}$$

$$\tilde{S}_{\mathrm{out}} = e^{i\phi}\tilde{S}_{\mathrm{in}} + ie^{i\phi/2}\sqrt{2\alpha_{\mathrm{l}}}a, \tag{2}$$

Here, we take the convention that the field varies in time as $\exp(-i\omega t)$. a is the amplitude of the SPP which is normalized such that $|a|^2$ corresponds to the time-averaged power along the $\tilde{z}$ direction. We note that $\tilde{S}_{\mathrm{in}}$ ($\tilde{S}_{\mathrm{out}}$) corresponds to the field distribution of the incident (reflected) light along the $\tilde{z}$ direction. Therefore, by expanding the incident (reflected) field into a series of plane waves as $\tilde{S}_{\mathrm{in(out)}} = \int_{-\infty}^{\infty} \tilde{s}_{\mathrm{in(out)}}(\tilde{k}_z) \exp(i\tilde{k}_z\tilde{z})d\tilde{k}_z$, the reflection coefficient for the incident plane wave case with a wavevector component $\tilde{k}_z$ is obtained as [1]

$$R(\tilde{k}_z) \equiv \frac{\tilde{s}_-}{\tilde{s}_+} = e^{i\phi}\frac{i(\tilde{k}_z - \beta_{\mathrm{spp}}) - \alpha_{\mathrm{l}} + \alpha_{\mathrm{spp}}}{i(\tilde{k}_z - \beta_{\mathrm{spp}}) + \alpha_{\mathrm{l}} + \alpha_{\mathrm{spp}}}. \tag{3}$$

We note that the $\tilde{z}$ coordinate defined on the metal surface is rather different from the x direction of the incident (reflected) beam profile coordinate, which is defined as perpendicular to the beam propagation direction. For the incident angle θ_0, the plane wave with the wavevector component k_x at the x direction has a $\tilde{z}$ component [3, 4]

$$\tilde{k}_z = k_x \cos\theta_0 + \beta_{\mathrm{spp}}, \left|\tilde{k}_z\right| < w, \tag{4}$$

where w is the angle spectrum width and assumed as $w\cos\theta_0 < \alpha_{\mathrm{l}} + |\alpha_{\mathrm{spp}}|$. Supplementary Eq. (4) can be understood as the central frequency of the angle spectrum shifting from 0

to β_{spp}. Such a spatial frequency shifting also happens to the reflected beam. Therefore, by substituting Supplementary Eq. (4) into (3), we obtain the transfer function during the reflection:

$$\begin{aligned} H(k_x) &= R(k_x \cos\theta_0 + \beta_{\text{spp}}) \\ &= e^{i\varphi} \frac{ik_x + A}{ik_x + B}, \end{aligned} \tag{5}$$

where $A = (\alpha_{\text{spp}} - \alpha_{\text{l}})/\cos\theta_0$ and $B = (\alpha_{\text{spp}} + \alpha_{\text{l}})/\cos\theta_0$.

Supplementary Note 2: Phase/Amplitude modulated field image generated by spatial light modulator

We generate the incident field images by a spatial light modulator (SLM: Holoeye PLUTO-NIR-011), which is a reflective-phase-only modulator. For the phase modulation, the system setup is schematically shown in Supplementary Fig. 2a, where a collimated laser beam was expanded and illuminated on the SLM and the phase-modulated image field was projected onto the metal film with a conjugate imaging system. In order to generate the amplitude-modulated incident field by the SLM, we used a Michelson configuration to make the phase-modulated field interfere with a reference plane wave (Supplementary Fig. 2b).

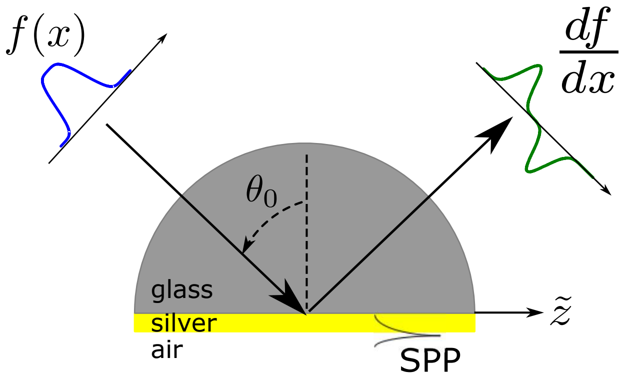

Supplementary Fig. 1. Schematic of a SPP reflector with the Kretschmann configuration Here, θ_0 corresponds to the incident angle where the phase-matching condition for SPP excitation is satisfied.

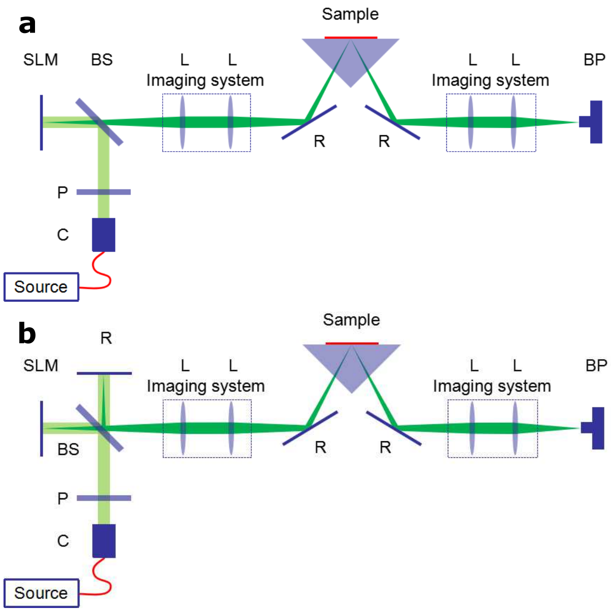

Supplementary Fig. 2. Schematic of optical system for the phase- and amplitude-modulation (a) and (b) correspond to phase- and amplitude-modulation, respectively. The field image is generated by spatial light modulator (SLM). Components include C: collimator, P: polarizer, BS: beam splitter, L: lens, SLM: spatial light modulator, R: reflector, BP: beam profiler.

Supplementary References

[1] Ruan, Z., Wu, H., Qiu, M. & Fan, S. Spatial control of surface plasmon polariton excitation at planar metal surface. *Opt. Lett.* **39**, 3587–3590 (2014).

[2] Lou, Y., Pan, H., Zhu, T. & Ruan, Z. Spatial coupled-mode theory for surface plasmon polariton excitation at metallic gratings. *J. Opt. Soc. Am. B* **33**, 819–824 (2016).

[3] Goodman, J. *Introduction to Fourier optics* 2nd edn (McGraw-hill, 1996).

[4] Doskolovich, L. L., Bykov, D. A., Bezus, E. A. & Soifer, V. A. Spatial differentiation of optical beams using phase-shifted Bragg grating. *Opt. Lett.* **39**, 1278–1281 (2014).
